# Characterizing the journey of Rett syndrome among females in the United States: a real-world evidence study using the Rett syndrome natural history study database

**DOI:** 10.1186/s11689-024-09557-6

**Published:** 2024-07-26

**Authors:** Damian May, Kalé Kponee-Shovein, Jeffrey L. Neul, Alan K. Percy, Malena Mahendran, Nathaniel Downes, Grace Chen, Talissa Watson, Dominique C. Pichard, Melissa Kennedy, Patrick Lefebvre

**Affiliations:** 1 Acadia Pharmaceuticals, San Diego, CA USA; 2 Analysis Group, Inc, Boston, MA USA; 3 Vanderbilt Kennedy Center, Nashville, TN USA; 4 Vanderbilt University Medical Center, Nashville, TN USA; 5 Heersink School of Medicine, The University of Alabama at Birmingham, Birmingham, AL USA; 6 International Rett Syndrome Foundation, Cincinnati, OH USA

**Keywords:** Rett syndrome, Disease progression, Natural history, Registry, Treatment patterns, Clinical manifestations

## Abstract

**Background:**

With the advent of the first targeted therapy for Rett Syndrome (RTT), a comprehensive assessment of the journey of RTT is needed to elucidate on present unmet needs in this population. This study characterized females with RTT in the United States and their disease journey with respect to longitudinal treatment patterns, RTT-related outcomes, and changes in disease severity.

**Methods:**

This retrospective cohort study used registry data of females with RTT from the 5211 RTT Natural History Study (RNHS) (November 2015–July 2021). Pharmacological and supportive therapy use, RTT-related outcomes, and RTT severity, as measured by the Clinical Severity Scale and Motor Behavioral Assessment scale, were evaluated following the first RNHS visit. Analyses were conducted overall and in subgroups by RTT type (classic and atypical RTT) and age at first visit (pediatric and adult).

**Results:**

A total of 455 females with RTT were included in the study, of whom 90.5% had classic RTT and 79.8% were pediatric individuals. Over a median follow-up of 4 years, use of pharmacological therapies, including prokinetic agents (42.7% vs. 28.3%), and supportive therapies, including physical therapy (87.3% vs. 40.2%) and speech-language therapy (86.8% vs. 23.9%), were more common in pediatric than adult individuals (all *p* < 0.05). Nearly half (44.6%) of all individuals had a hospital or emergency room visit, with a higher proportion of visits in individuals with classic RTT than atypical RTT and pediatric than adult individuals (both *p* = 0.001). An increasing trend in clinical severity was observed in pediatric individuals (mean change per year: 0.24; 95% confidence interval [CI]: 0.03, 0.44), while an increasing trend in motor-behavioral dysfunction was observed in pediatric individuals (mean change per year: 1.12; 95% CI: 0.63, 1.60) and those with classic RTT (mean change per year: 0.97; 95% CI: 0.53, 1.41).

**Conclusions:**

Findings from this study highlight the considerable burden of RTT across disease subtype and age. Despite reliance on supportive therapies and healthcare encounters, individuals with RTT experience increasing disease severity and motor-behavioral dysfunction in childhood and adolescence, underscoring the unmet needs of this population and the value of early intervention to manage RTT in the long-term.

**Supplementary Information:**

The online version contains supplementary material available at 10.1186/s11689-024-09557-6.

## Background

Rett syndrome (RTT) is a severe neurodevelopmental disorder that occurs almost exclusively in females, with an estimated incidence of 1 out of every 10,000 to 15,000 live female births worldwide [1–3]. RTT is clinically diagnosed as classic or atypical based on the manifestation of key clinical symptoms [2], with an average age at diagnosis of 2.5 years [2]. In 90–95% of individuals with classic RTT, the disorder is caused by a spontaneous mutation in the *MECP2* gene on the X chromosome [2, 3]. There are four main criteria required for diagnosing classic RTT, including the partial or complete loss of acquired purposeful hand skills, the partial or complete loss of spoken language, stereotypic hand movements, and gait abnormalities [2]. At least two of the four main criteria are required to diagnose atypical RTT, in addition to at least five of 11 supportive criteria [2, 4]. 

Despite an estimated survival of over 70% at 45 years of age [5], RTT is associated with substantial clinical and humanistic burden that translates to poor quality of life (QoL) [2, 6]. Individuals with RTT often require lifelong care due to a range of symptoms, stemming from neurological, gastrointestinal, cardiac, endocrine, and orthopedic disorders [5, 7]. However, there is limited longitudinal data describing how the severity of RTT changes over time, underscoring a much-needed area of research.

Until recently, treatment options for RTT were limited to symptom management and supportive care for daily activities [2, 4, 8]. To promote childhood development, consensus guidelines recommend early referral to physical, occupational, and speech language therapists, as well as establishment of an individualized education program [2]. Anticonvulsants may be used to treat seizures, and maintaining a healthy body mass and monitoring for scoliosis become important considerations as individuals reach late childhood [2]. 

In March 2023, the United States (US) Food and Drug Administration (FDA) approved trofinetide as the first pharmaceutical therapy for RTT [9, 10]. With the advent of trofinetide for treatment of RTT, a comprehensive understanding of the characteristics and disease journey of individuals with RTT is needed to elucidate on present unmet needs and inform the integration of novel therapies into the current treatment paradigm for RTT.

The overarching aim of this study was to characterize females with classic or atypical RTT in the US with respect to their demographic and clinical profiles, and to describe their disease journey with respect to longitudinal patterns of treatment, RTT-related outcomes, and changes in disease severity.

## Methods

### Data source

All analyses were conducted using registry data from the 5211 RTT Natural History Study (RNHS) spanning from November 2015 to July 2021. The 5211 RNHS is a US-based, multi-center, five-year observational registry study that has been comprehensively described in prior literature [5, 6, 11–14]. Briefly, it includes data on measures such as demographics, developmental skills (clinician-recorded or caregiver-reported), RTT clinical features, supportive therapy (e.g., physical therapy), medication logs, and death, as well as clinical severity and motor-behavioral dysfunction, as evaluated by the Clinical Severity Scale (CSS) and Motor Behavioral Assessment (MBA) scales, respectively. Data were de-identified and compliant with the Health Insurance Portability and Accountability Act.

### Study design and population

A retrospective, longitudinal cohort design was used to address the study objectives. The index date was defined as the date of the first RNHS visit. The follow-up period was defined as the time after the index date up to the earliest date of study disenrollment, death, or study completion.

The study population consisted of females with a diagnosis of classic or atypical RTT, at least one follow-up visit, and no history of brain trauma on or before the index date. The overall study sample was further stratified into subgroups by RTT type (classic RTT and atypical RTT) and age at index (pediatric [< 18 years of age]) and adult [≥ 18 years of age]).

### Outcomes

Demographics and clinical characteristics were evaluated on the index date and included age at first visit, age at onset of regression, race, *MECP2* mutation status, clinical manifestations of RTT, and gross motor function.

Pharmacological and supportive therapy use were evaluated during the follow-up period. Pharmacological therapies included the use of prokinetic agents, antiepileptics, sedative/hypnotics, and nutritional supplements. Reasons for discontinuation of pharmacologic therapy, including ineffective treatment, treatment not needed, and side effects, were reported among individuals who discontinued treatment. Supportive therapies included the use of physical therapy, speech-language therapy, occupational therapy, scoliosis treatment (including bracing, serial casting, and surgery), behavioral therapy, vision therapy, and feeding assistance (including feeding tube). Feeding assistance was identified from hospital or emergency room visits that listed feeding assistance as the reason for the visit.

RTT-related outcomes were evaluated during the follow-up period and included hospital and emergency room visits, incident gastrostomy tube (g-tube) surgery, and death. Incident g-tube surgery was evaluated among individuals without a prior g-tube surgery on the index date.

RTT severity was assessed based on the CSS and MBA scale and evaluated at each year following the first visit. The CSS is a clinician-completed questionnaire that uses a Likert-type scale to rank statements in 13 categories related to the features of RTT for a maximum score of 58 [15]. The MBA scale is a clinician-completed questionnaire that uses a Likert-type scale to score 34 items on a severity scale of 0 to 4 (none, 25% of the time, 50% of the time 75% of the time, 100% of the time) for a maximum total score of 136 [13]. For both the CSS and MBA scale, higher scores represent greater clinical severity.

### Statistical analysis

All statistical analyses were conducted using SAS Enterprise 7.1 software (SAS Institute, Cary, NC, USA). The extent of missing data was summarized for all study measures. All measures in this study were evaluated in the subset of individuals with complete information on these measures. No imputation was conducted for missing data. Demographics, clinical characteristics, use of pharmacological and supportive therapies, RTT-related outcomes, and annual change in RTT severity were summarized using means, standard deviations (SDs), and medians for continuous characteristics and frequencies and proportions for categorical characteristics.

Due to the substantial missingness (> 65%) in CSS and MBA scores from years 3–5 of follow up, these measures were only evaluated from the first RNHS visit to year 2 of follow-up. CSS and MBA scores were summarized for each respective year among individuals with available measurements using means SDs, and medians. Separate linear mixed effect models were used to estimate mean change in CSS and MBA scores per year and corresponding 95% confidence intervals (CIs).

Statistical comparisons between individuals with classic RTT vs. atypical RTT and pediatric individuals vs. adult individuals were conducted using t-tests for means and Chi-squared tests or Fisher’s exact tests (when the expected sample size was < 5) for proportions. The t-statistic was reported for comparisons conducted using the t-test and the Chi-squared value was reported for comparisons conducted using the Chi-squared test or Fisher’s exact test. Nominal *p-*values < 0.05 were considered statistically significant and were not adjusted for multiple comparisons.

## Results

### Study population

After applying all eligibility criteria, 455 females with RTT were included in the study, of whom 412 (90.5%) had classic RTT and 43 (9.5%) had atypical RTT. Three hundred and sixty-three individuals (79.8%) were pediatric, while 92 (20.2%) were adults.

Demographic and clinical characteristics of the study population are presented in Table 1. Mean (SD) age at first visit was 11.8 (9.5) years in the overall RTT cohort, 7.9 (4.7) years among pediatric individuals, and 27.1 (8.1) years among adult individuals. Mean (SD) age of motor and communication regression was 2.3 (0.8) years overall. Individuals primarily identified as White (87.0%) and nearly all had an *MECP2* mutation (98.2%).

**Table 1:** Demographics and clinical characteristics among females with RTT, overall and stratified by RTT type and age

Characteristics	Overall RTT Cohort	Stratification by RTT type				Stratification by age			
		Classic RTT	Atypical RTT	Test statistic	*p*-value	Pediatric (< 18 years of age)	Adult (≥ 18 years of age)	Test statistic	*p*-value
	(*N* = 455)	(*N* = 412)	(*N* = 43)			(*N* = 363)	(*N* = 92)		
**Demographics**									
Age at first visit, years, mean ± SD [median]	11.8 ± 9.5 [9]	11.8 ± 9.5 [9]	11.7 ± 9.7 [9]	-0.1	0.943	7.9 ± 4.7 [7]	27.1 ± 8.1 [25]	21.9	< 0.001*
*Age category at first visit*,* n (%)*									
0–4	110 (24.2)	98 (23.8)	12 (27.9)	0.4	0.548	110 (30.3)	–	–	–
5–10	149 (32.7)	134 (32.5)	15 (34.9)	0.1	0.754	149 (41.0)	–	–	–
11–17	104 (22.9)	97 (23.5)	7 (16.3)	1.2	0.280	104 (28.7)	–	–	–
18–29	61 (13.4)	56 (13.6)	5 (11.6)	0.1	0.719	–	61 (66.3)	–	–
30–39	25 (5.5)	21 (5.1)	4 (9.3)	1.3	0.280	–	25 (27.2)	–	–
40–49	5 (1.1)	5 (1.2)	0 (0.0)	0.5	1.000	–	5 (5.4)	–	–
≥ 50	1 (0.2)	1 (0.2)	0 (0.0)	0.1	1.000	–	1 (1.1)	–	–
Age of regression onset, years, mean ± SD [median]	2.3 ± 0.8 [2]	2.4 ± 0.8 [2]	1.9 ± 1.2 [2]	-2.4	0.021*	2.4 ± 0.8 [2]	2.2 ± 0.8 [2]	-1.5	0.143
*Race*,* n (%)*									
White	396 (87.0)	357 (86.7)	39 (90.7)	0.6	0.452	310 (85.4)	86 (93.5)	4.2	0.039*
Multiple	21 (4.6)	20 (4.9)	1 (2.3)	0.6	0.709	20 (5.5)	1 (1.1)	3.3	0.093
Black or African American	15 (3.3)	14 (3.4)	1 (2.3)	0.1	1.000	12 (3.3)	3 (3.3)	0.0	1.000
Asian	14 (3.1)	14 (3.4)	0 (0.0)	1.5	0.381	13 (3.6)	1 (1.1)	1.5	0.319
Native Hawaiian and Other Pacific Islander	1 (0.2)	1 (0.2)	0 (0.0)	0.1	1.000	1 (0.3)	0 (0.0)	0.3	1.000
Unknown/unspecified	8 (1.8)	6 (1.5)	2 (4.7)	2.3	0.170	7 (1.9)	1 (1.1)	0.3	1.000
**Clinical characteristics**									
*MECP2 mutation*,* n (%)*	447 (98.2)	406 (98.5)	41 (95.3)	2.3	0.170	358 (98.6)	89 (96.7)	1.5	0.206
*Clinical manifestations*,* n (%)*									
Loss of spoken language	436 (95.8)	410 (99.5)	26 (60.5)	148.4	< 0.001*	348 (95.9)	88 (95.7)	0.0	1.000
Hand stereotypies	420 (92.3)	389 (94.4)	31 (72.1)	27.3	< 0.001*	339 (93.4)	81 (88.0)	3.0	0.086
Respiratory dysfunction	345 (75.8)	326 (79.1)	19 (44.2)	25.9	< 0.001*	281 (77.4)	64 (69.6)	2.5	0.116
Sleep disturbances	344 (75.6)	318 (77.2)	26 (60.5)	5.9	0.015*	272 (74.9)	72 (78.3)	0.4	0.507
Constipation	339 (74.5)	308 (74.8)	31 (72.1)	0.1	0.703	262 (72.2)	77 (83.7)	5.1	0.024*
Feeding problems	292 (64.2)	268 (65.0)	24 (55.8)	1.4	0.229	233 (64.2)	59 (64.1)	0.0	0.992
Autonomic symptoms	274 (60.2)	248 (60.2)	26 (60.5)	0.0	0.972	216 (59.5)	58 (63.0)	0.4	0.536
Scoliosis	234 (51.4)	217 (52.7)	17 (39.5)	2.7	0.101	166 (45.7)	68 (73.9)	23.3	< 0.001*
Gastroesophageal reflux	212 (46.6)	198 (48.1)	14 (32.6)	3.8	0.053	164 (45.2)	48 (52.2)	1.4	0.230
Epilepsy	210 (46.2)	196 (47.6)	14 (32.6)	3.5	0.060	158 (43.5)	52 (56.5)	5.0	0.026*
Fractures	17 (3.7)	14 (3.4)	3 (7.0)	1.4	0.210	12 (3.3)	5 (5.4)	0.9	0.356
Gall bladder dysfunction	8 (1.8)	8 (1.9)	0 (0.0)	0.8	1.000	3 (0.8)	5 (5.4)	9.0	0.010*
*Gross motor function*,* n (%)*									
Ability to sit	339 (74.5)	305 (74.0)	34 (79.1)	0.5	0.470	277 (76.3)	62 (67.4)	3.1	0.080
Ability to stand	223 (49.0)	198 (48.1)	25 (58.1)	1.6	0.208	177 (48.8)	46 (50.0)	0.0	0.832
Ambulation	220 (48.4)	195 (47.3)	25 (58.1)	1.8	0.177	173 (47.7)	47 (51.1)	0.3	0.557
Ability to communicate	34 (7.5)	31 (7.5)	3 (7.0)	0.0	1.000	26 (7.2)	8 (8.7)	0.2	0.617

Abbreviations: RTT: Rett syndrome; SD: standard deviation

Asterisks denote statistical significance at p<0.05

Common clinical manifestations of RTT included loss of language (95.8%), hand stereotypies (92.3%), respiratory dysfunction (75.8%), sleep disturbances (75.6%), and constipation (74.5%). Clinical manifestations were more prevalent in individuals with classic RTT than atypical RTT (loss of language: 99.5% vs. 60.5%, *p <* 0.001; hand stereotypies: 94.4% vs. 72.1%, *p <* 0.001; respiratory dysfunction: 79.1% vs. 44.2%, *p* < 0.001; sleep disturbances: 77.2% vs. 60.5%, *p* = 0.015; respectively). At first visit, scoliosis (73.9% vs. 45.7%, *p* < 0.001), constipation (83.7% vs. 72.2%, *p* = 0.024), and epilepsy (56.5% vs. 43.5%, *p* = 0.026) were more prevalent in adult individuals than pediatric individuals, respectively. There were no significant differences in ability to sit, stand, or walk independently between individuals with classic RTT and atypical RTT (classic: 47.3–74.0%; atypical: 58.1–79.1%).

### Use of pharmacological and supportive therapies during the follow-up period

Overall, the most used pharmacological therapies were prokinetic agents (39.8%) and antiepileptic drugs (32.3%) (Table 2). Compared with adult individuals, pediatric individuals were more likely to use prokinetic agents (42.7% vs. 28.3%, *p* = 0.011), antiepileptics (35.0% vs. 21.7%, *p* = 0.015), sedatives/hypnotics (27.8% vs. 15.2%, *p* = 0.013), and nutritional supplements (25.1% vs. 15.2%, *p* = 0.045) (Table 2). Use of pharmacological therapies was similar between individuals with classic RTT and individuals with atypical RTT (Table 2).

**Table 2:** Pharmacological therapies used among females with RTT, overall and stratified by RTT type and age

Pharmacological therapies	Overall RTT Cohort	Stratification by RTT type				Stratification by age			
		Classic RTT	Atypical RTT	Test statistic	*p*-value	Pediatric (< 18 years of age)	Adult (≥ 18 years of age)	Test statistic	*p*-value
	(*N* = 455)	(*N* = 412)	(*N* = 43)			(*N* = 363)	(*N* = 92)		
**Follow-up period**,** years**,** mean ± SD [median]**	4.1 ± 1.0 [4]	4.1 ± 1.0 [4]	4.1 ± 1.2 [4]	-0.2	0.833	4.1 ± 1.1 [4]	4.2 ± 0.8 [4]	1.5	0.140
Prokinetic agents, n (%)	181 (39.8)	164 (39.8)	17 (39.5)	0.0	0.972	155 (42.7)	26 (28.3)	6.4	0.011*
Individuals who discontinued use	46 (25.4)	44 (26.8)	2 (11.8)	1.8	0.246	43 (27.7)	3 (11.5)	3.1	0.079
*Reasons for discontinuing*^*1*^									
Ineffective	11 (23.9)	10 (22.7)	1 (50.0)	0.8	0.425	11 (25.6)	0 (0.0)	1.0	1.000
Not needed	29 (63.0)	28 (63.6)	1 (50.0)	0.2	1.000	28 (65.1)	1 (33.3)	1.2	0.545
Side effects	10 (21.7)	10 (22.7)	0 (0.0)	0.6	1.000	8 (18.6)	2 (66.7)	3.8	0.115
Missing	2 (4.3)	2 (4.5)	0 (0.0)	0.1	1.000	2 (4.7)	0 (0.0)	0.1	1.000
Antiepileptics, n (%)	147 (32.3)	137 (33.3)	10 (23.3)	1.8	0.182	127 (35.0)	20 (21.7)	5.9	0.015*
Individuals who discontinued use	41 (27.9)	40 (29.2)	1 (10.0)	1.7	0.284	36 (28.3)	5 (25.0)	0.1	0.756
*Reasons for discontinuing*^*1*^									
Ineffective	20 (48.8)	20 (50.0)	0 (0.0)	1.0	1.000	17 (47.2)	3 (60.0)	0.3	0.663
Not needed	10 (24.4)	10 (25.0)	0 (0.0)	0.3	1.000	9 (25.0)	1 (20.0)	0.1	1.000
Side effects	13 (31.7)	12 (30.0)	1 (100.0)	2.2	0.317	12 (33.3)	1 (20.0)	0.4	1.000
Missing	3 (7.3)	3 (7.5)	0 (0.0)	0.1	1.000	3 (8.3)	0 (0.0)	0.4	1.000
Sedatives/hypnotics, n (%)	115 (25.3)	101 (24.5)	14 (32.6)	1.3	0.248	101 (27.8)	14 (15.2)	6.2	0.013*
Individuals who discontinued use	25 (21.7)	23 (22.8)	2 (14.3)	0.5	0.731	23 (22.8)	2 (14.3)	0.5	0.731
*Reasons for discontinuing*^*1*^									
Ineffective	9 (36.0)	8 (34.8)	1 (50.0)	0.2	1.000	8 (34.8)	1 (50.0)	0.2	1.000
Not needed	11 (44.0)	10 (43.5)	1 (50.0)	0.0	1.000	11 (47.8)	0 (0.0)	1.7	0.487
Side effects	9 (36.0)	8 (34.8)	1 (50.0)	0.2	1.000	7 (30.4)	2 (100.0)	3.9	0.120
Missing	1 (4.0)	1 (4.3)	0 (0.0)	0.1	1.000	1 (4.3)	0 (0.0)	0.1	1.000
Nutritional supplements, n (%)	105 (23.1)	95 (23.1)	10 (23.3)	0.0	0.977	91 (25.1)	14 (15.2)	4.0	0.045*
Individuals who discontinued use	20 (19.0)	19 (20.0)	1 (10.0)	0.6	0.683	20 (22.0)	0 (0.0)	3.8	0.067
*Reasons for discontinuing*^*1*^									
Ineffective	1 (5.0)	1 (5.3)	0 (0.0)	0.1	1.000	1 (5.0)	0 (0.0)	–	–
Not needed	16 (80.0)	15 (78.9)	1 (100.0)	0.3	1.000	16 (80.0)	0 (0.0)	–	–
Side effects	3 (15.0)	2 (10.5)	1 (100.0)	6.0	0.150	3 (15.0)	0 (0.0)	–	–
Missing	1 (5.0)	1 (5.3)	0 (0.0)	0.1	1.000	4 (15.0)	0 (0.0)	–	–

Abbreviations: RTT: Rett syndrome; SD: standard deviation

Note:

1. Individuals could have multiple reasons for discontinuing a treatment

Asterisks denote statistical significance at p<0.05

The most common reason for discontinuing prokinetic agents and sedatives/hypnotics among individuals who discontinued treatment was that the therapy was no longer needed (63.0% and 44.0%, respectively) (Table 2). Among individuals who discontinued antiepileptic drugs, nearly half (48.8%) stopped use due to ineffective treatment (Table 2).

**Fig. 1:**
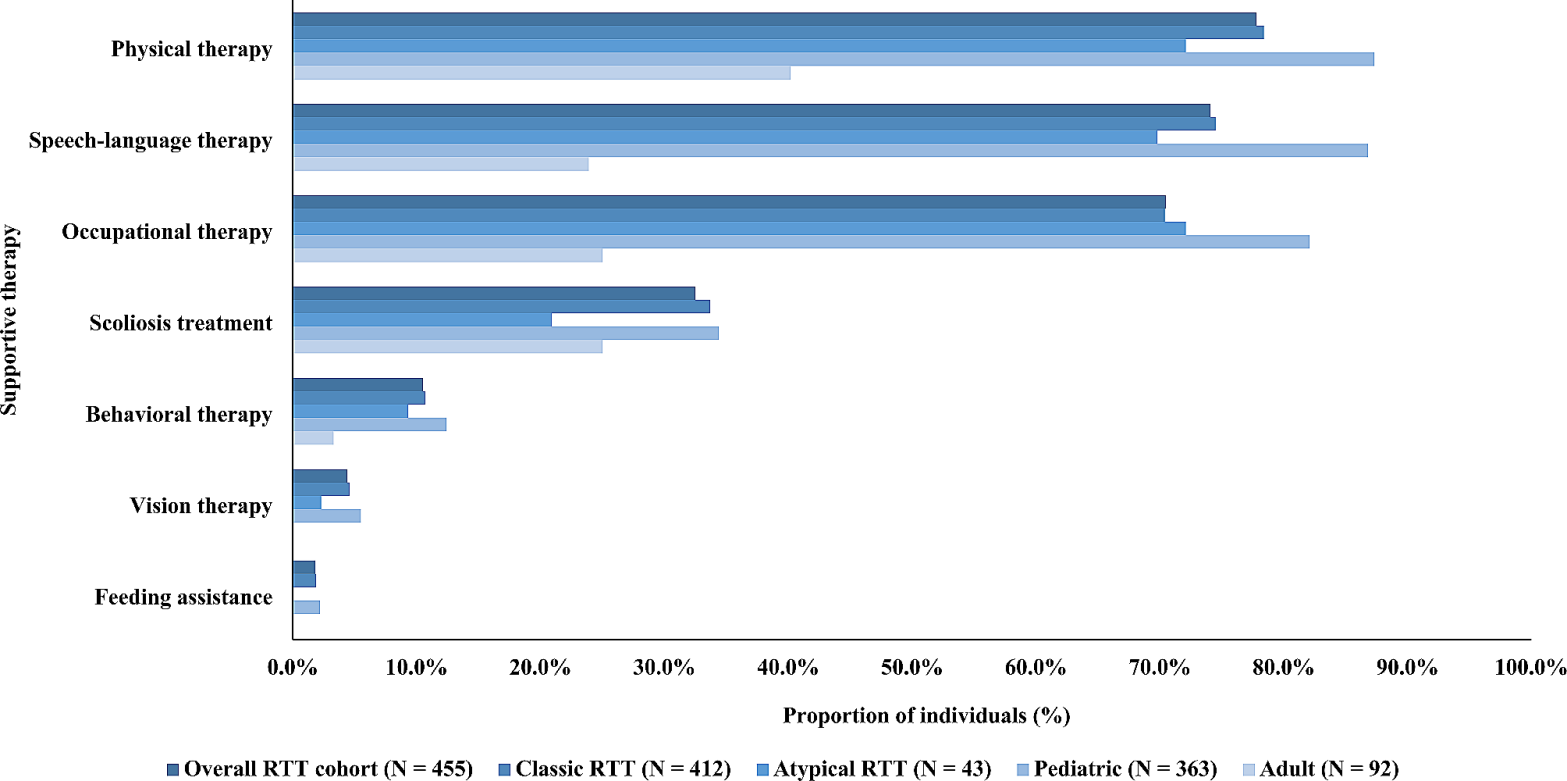
Supportive therapies used among females with RTT, overall and stratified by RTT type and age Abbreviation: RTT: Rett syndrome

The most used supportive therapies were physical therapy (77.8%), speech-language therapy (74.1%), and occupational therapy (70.5%) (Fig. 1). Pediatric individuals were more likely than adult individuals to use physical therapy (87.3% vs. 40.2%, *p* < 0.001), speech-language therapy (86.8% vs. 23.9%, *p* < 0.001), and occupational therapy (82.1% vs. 25.0%, *p* < 0.001) (Supplementary Table 1). Use of supportive therapies was similar between individuals with classic RTT and atypical RTT (classic: 1.9–78.4%; atypical: 0.0–72.1%) (Supplementary Table 1).

### RTT-related outcomes during the follow-up period

**Fig. 2:**
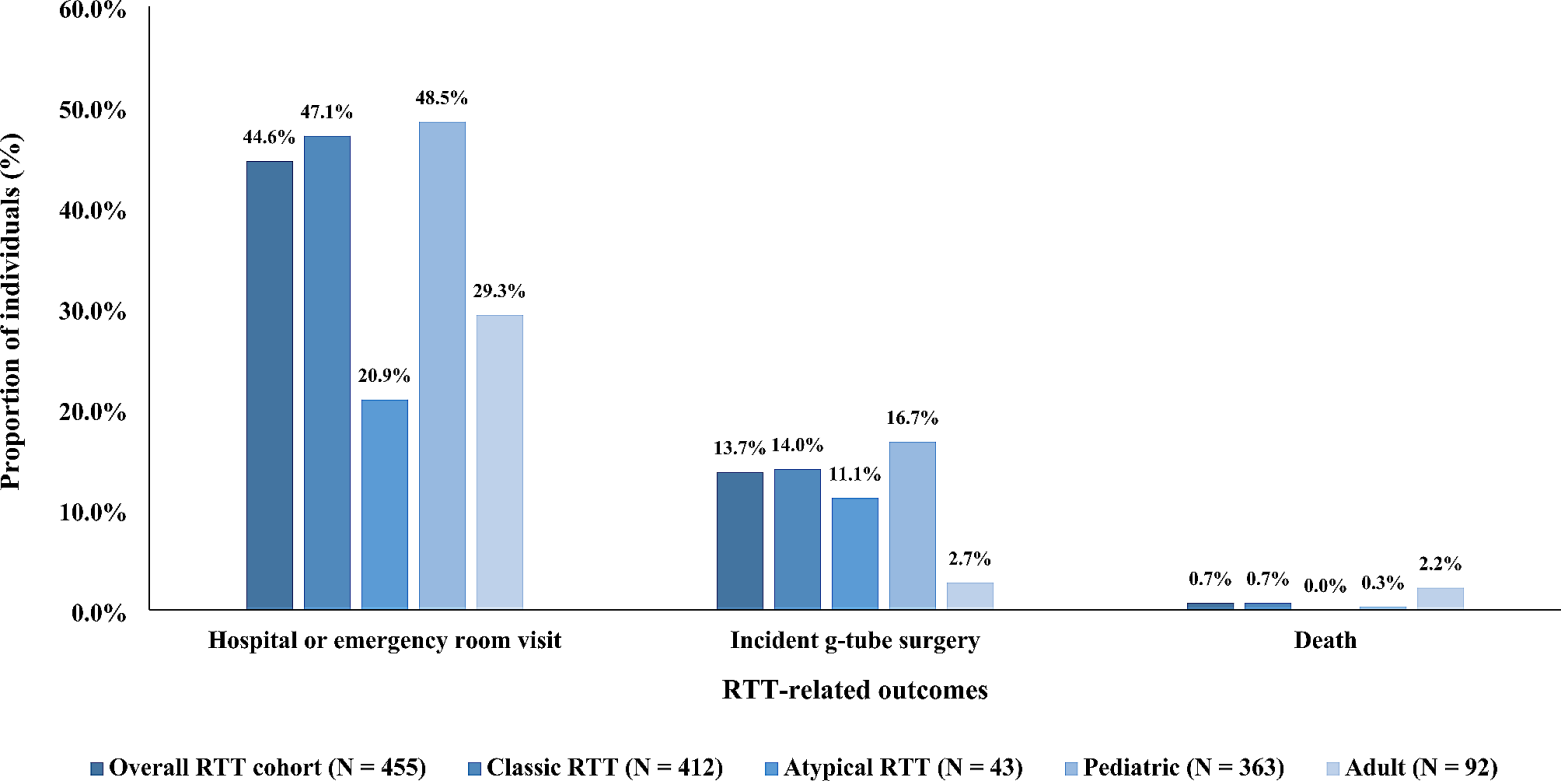
RTT-related outcomes among females with RTT, overall and stratified by RTT type and age^1,2^ Abbreviations: G-tube: gastrostomy tube; RTT: Rett syndrome. 1. Proportions of individuals with incident g-tube surgery were evaluated among individuals without a g-tube surgery prior to the first visit (overall cohort: *N* = 350; classic RTT: *N* = 314; atypical RTT: *N* = 36; pediatric: *N* = 275; adult: *N* = 75). 2. G-tube surgeries included endoscopic gastrostomy, gastrostomy with fundoplication, and gastrostomy without fundoplication

Over a median follow-up period of 4 years, nearly half (44.6%) of all individuals had a hospital or emergency room visit (Fig. 2), with a significantly higher proportion of visits observed in individuals with classic RTT than atypical RTT (47.1% vs. 20.9%, *p* = 0.001), and pediatric individuals than adult individuals (48.5% vs. 29.3%, *p* = 0.001) (Supplementary Table 2). Incident g-tube surgeries were observed among 13.7% of individuals, and pediatric individuals had a significantly higher incidence of g-tube surgery than adult individuals (16.7% vs. 2.7%, *p* = 0.002) (Supplementary Table 2). Mortality was rare (0.7%) in the overall RTT cohort, and all observed deaths were due to natural causes, with no specific cause documented (Fig. 2).

### Change in CSS and MBA scores from first visit to year 2 of follow-up

**Fig. 3:**
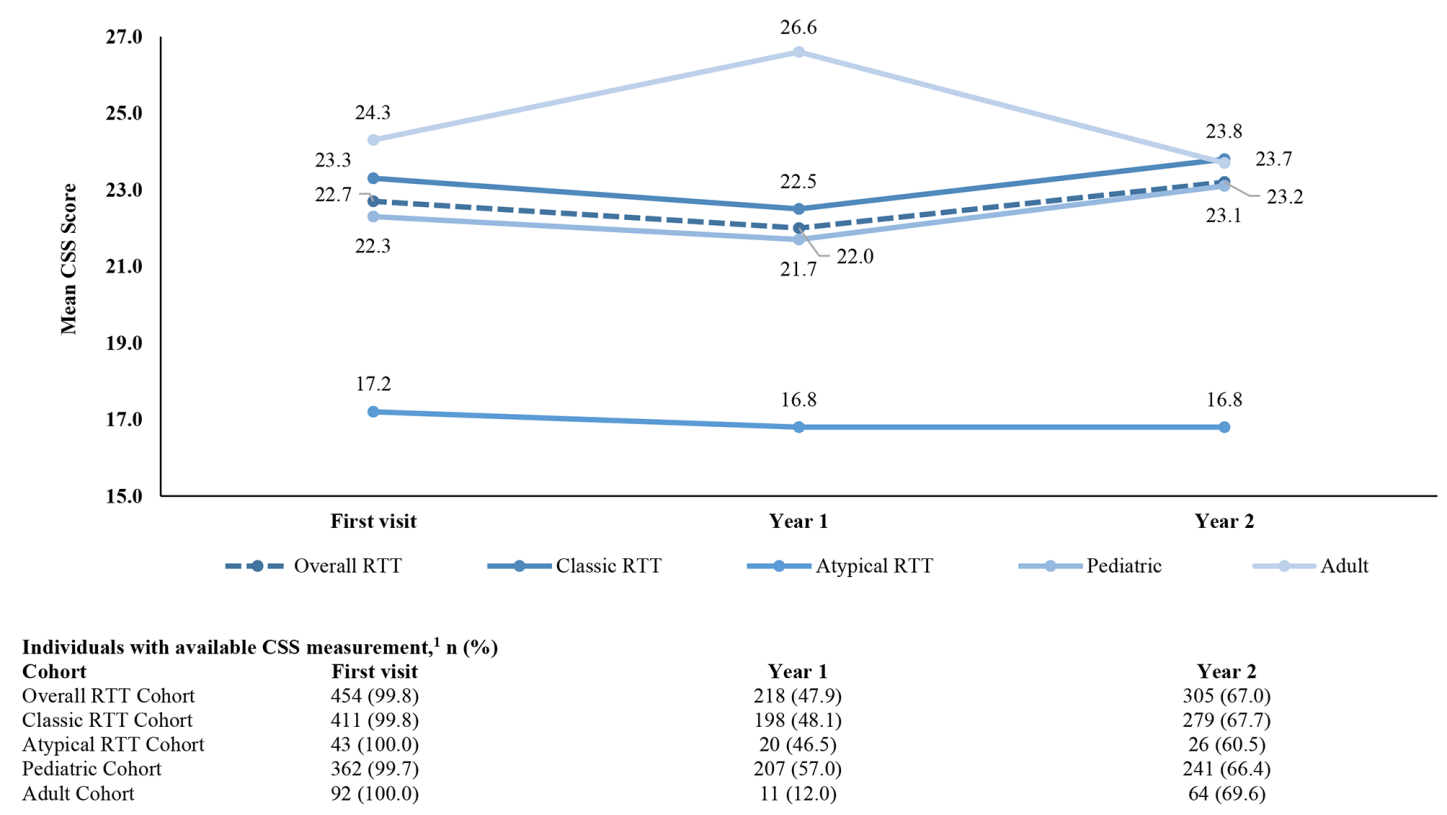
Trends in CSS score among females with RTT, overall and stratified by RTT type and age Abbreviations: CSS: Clinical Severity Scale; RTT: Rett syndrome. Note: 1. CSS score per year was calculated only among individuals with an available CSS measurement in the respective year

**Fig. 4:**
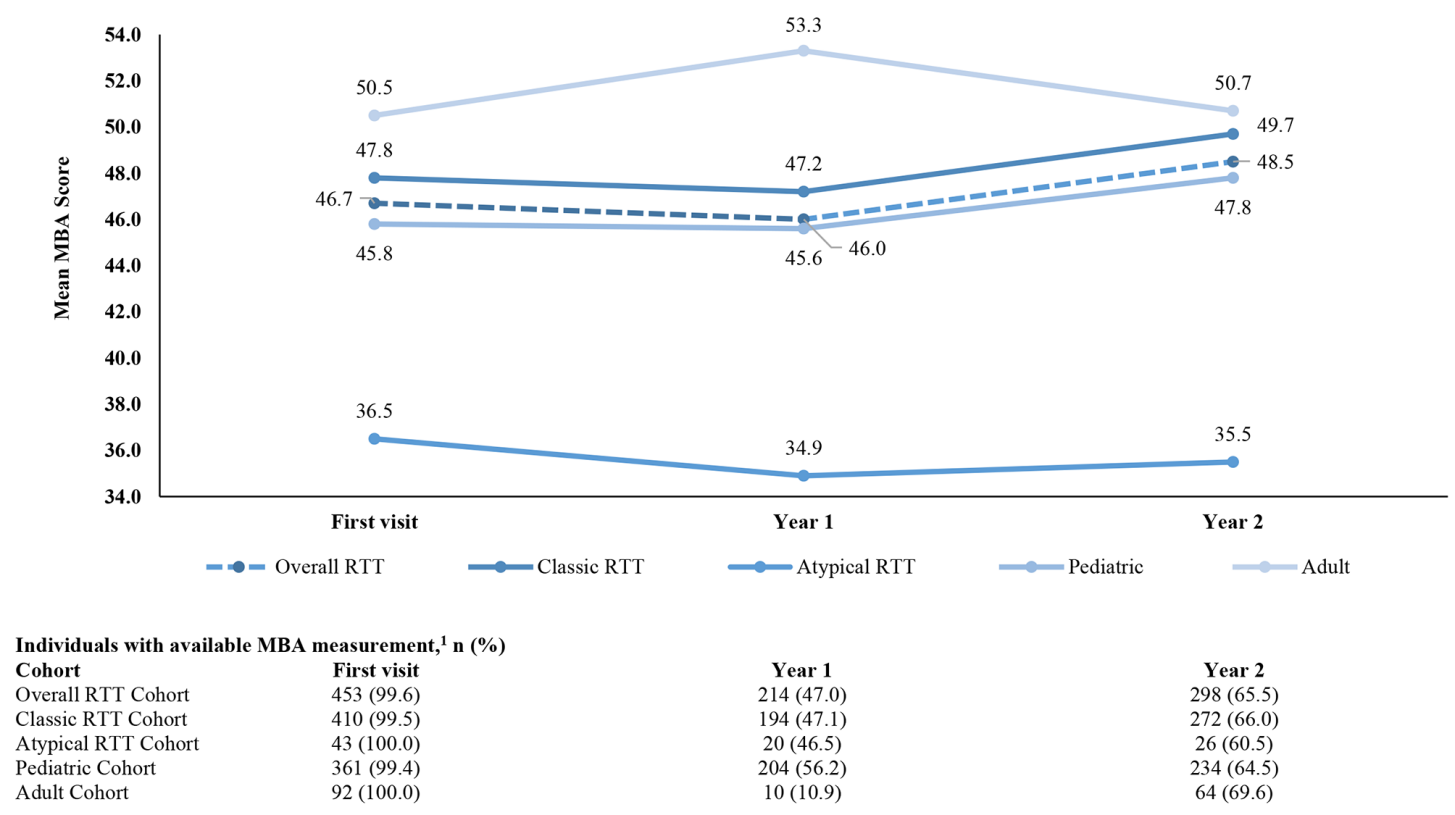
Trends in MBA score among females with RTT, overall and stratified by RTT type and age Abbreviations: MBA: Motor Behavioral Assessment; RTT: Rett syndrome. Note: 1. MBA score per year was calculated only among individuals with an available MBA measurement in the respective year

In the overall RTT cohort, mean CSS score (first visit: 22.7; year 1: 22.0; year 2: 23.2) (Fig. 3) and mean MBA score (first visit: 46.7; year 1: 46.0; year 2: 48.5) (Fig. 4) remained largely unchanged from first visit to year 2 of follow up. Mean CSS scores were significantly higher for individuals with classic RTT than individuals with atypical RTT across all timepoints assessed (classic: 22.5‒23.8; atypical: 16.8‒17.2, all *p* < 0.05) (Supplementary Table 3); a similar pattern was observed for mean MBA scores (classic: 47.2‒49.7; atypical: 34.9‒36.5, all *p* < 0.05) (Supplementary Table 4). Mean CSS score was significantly higher for adult individuals than pediatric individuals at year 1 of follow-up (adult: 26.6; pediatric: 21.7, *p* = 0.031) (Supplementary Table 3), while mean MBA scores were significantly higher for adult individuals than pediatric individuals at first visit (adult: 50.5; pediatric: 45.8, *p* = 0.009) and year 1 of follow-up (adult: 53.3; pediatric: 45.6, *p* = 0.046) (Supplementary Table 4).

There were no meaningful changes in clinical severity per year in the overall RTT cohort (mean change per year: 0.11; 95% CI: -0.08, 0.30), adult individuals (mean change per year: -0.39; 95% CI: -0.83, 0.05), individuals with classic RTT (mean change per year: 0.12; 95% CI: -0.07, 0.31), or individuals with atypical RTT (mean change per year: 0.00; 95% CI: -0.70, 0.69) (Table 3). Conversely, an increasing trend in clinical severity per year was observed in pediatric individuals (mean change per year: 0.24; 95% CI: 0.03, 0.44) (Table 3).

**Table 3:** Annual change in CSS and MBA scores among females with RTT, overall and stratified by RTT type and age

Clinical severity measures	Overall RTT Cohort	Stratification by RTT type		Stratification by age	
		Classic RTT	Atypical RTT	Pediatric (< 18 years of age)	Adult (≥ 18 years of age)
	(*N* = 455)	(*N* = 412)	(*N* = 43)	(*N* = 363)	(*N* = 92)
**Follow-up period**,** years**,** mean ± SD [median]**	4.1 ± 1.0 [4]	4.1 ± 1.0 [4]	4.1 ± 1.2 [4]	4.1 ± 1.1 [4]	4.2 ± 0.8 [4]
Mean change in CSS per year (95% CI)	0.11 (-0.08, 0.30)	0.12 (-0.07, 0.31)	0.00 (-0.70, 0.69)	0.24 (0.03, 0.44)	-0.39 (-0.83, 0.05)
Mean change in MBA score per year (95% CI)	0.92 (0.50, 1.35)	0.97 (0.53, 1.41)	0.34 (-1.06, 1.75)	1.12 (0.63, 1.60)	0.17 (-0.61, 0.94)

Abbreviations: CI: confidence interval; CSS: Clinical Severity Scale; MBA: Motor Behavioral Assessment; RTT: Rett syndrome; SD: standard deviation

An increasing trend in motor-behavioral dysfunction (as evaluated using the MBA scale) per year was observed in the overall RTT cohort (mean change per year: 0.92; 95% CI: 0.50, 1.35), pediatric individuals (mean change per year: 1.12; 95% CI: 0.63, 1.60), and individuals with classic RTT (mean change per year: 0.97; 95% CI: 0.53, 1.41) (Table 3). No meaningful changes in motor-behavioral dysfunction per year were observed in adult individuals (mean change per year: 0.17; 95% CI: -0.61, 0.94) or individuals with atypical RTT (mean change per year: 0.34; 95% CI: -1.06, 1.75) (Table 3).

## Discussion

In this retrospective real-world study, we used registry data from the 5211 RHNS study to gain insights into the disease journey of females with RTT in the US with respect to longitudinal treatment patterns, RTT-related outcomes, and changes in disease severity. Our findings suggest that individuals with RTT experience high disease burden, irrespective of RTT type and age group, as evidenced by the high use of supportive therapies, the need for a hospital or emergency room visit in nearly half of all individuals, and the increasing trend in motor-behavioral dysfunction observed over time. To our knowledge, this is the first real-world study to evaluate changes in RTT severity over time, as measured by the CSS and MBA scale. These findings help advance the understanding of short-term changes in RTT severity across RTT type and age group.

In this study, most individuals with RTT required supportive therapy, with more use observed in pediatric individuals than adult individuals. These findings are consistent with a recent real-world administrative healthcare claims study of females with RTT, which found that nearly 60% of the overall RTT cohort relied on pharmacologic and supportive therapies to manage symptoms, and the use of supportive therapies was highest during early childhood (3–4 years of age) and decreased markedly by 18 years of age [16]. The most used pharmacological therapies in this study were prokinetic agents and antiepileptic drugs, which aligns with the high rates of gastrointestinal manifestations and epilepsy reported in this population. In one survey of 983 parents of females with RTT in the North American RTT database, 92% reported symptoms of gastrointestinal dysmotility [17], while epilepsy has been estimated to occur in 60–80% of individuals with RTT [18]. As the registry data used in this study spanned up to July 2021, prior to the FDA approval of trofinetide in March 2023 [9, 10], future real-world evidence studies that encompass data following the approval of trofinetide or other novel therapies are warranted to shed light on its integration into the treatment paradigm for RTT, and its impact on pharmacologic and supportive therapies currently used to manage RTT symptoms.

Despite the heavy reliance on pharmacologic and supportive therapies, nearly half of all individuals in our study required a hospital or emergency room visit during the follow-up period. While comparable literature is limited, a previous survey study of 399 individuals with RTT from the International Rett Syndrome Phenotype Database (InterRett) database (95.5% female and 82.2% from the US) found that 21.4% of individuals experienced a hospital admission for lower respiratory tract infection (LRTI) over the previous 5 years [19], a figure that is likely lower due to the capture of LRTI-related hospital admissions rather than all-cause hospital or emergency room visits. Moreover, a higher frequency of hospital or emergency room visits was observed in individuals with classic RTT relative to atypical RTT and in pediatric individuals relative to adult individuals. A registry-based study in Australia similarly observed a higher frequency of hospital admissions in individuals with RTT between the ages of 0–17 years relative to individuals with RTT more than 17 years of age (0–7 years: 22.2%, 8–12 years: 23.7%, 13–17 years: 18.0%, > 17 years: 9.1%) [20], although these findings are not directly comparable to our frequency estimate given that emergency room visits could not be distinguished from hospital visits in this study. As the present study was unable to separately assess the frequency of emergency room visits and hospital visits, an understanding of the severity of healthcare encounters experienced by individuals in this study is limited.

In this study, approximately 14% of individuals with RTT underwent incident g-tube surgery during the follow-up period, with a greater incidence in pediatric individuals than adult individuals. Although literature on the incidence of g-tube surgery in individuals with RTT is sparse, prior literature have reported a prevalence of g-tube surgery between 28.0 and 30.3% [17, 21], which aligns with the prevalence of g-tube surgery that can be delineated from our study given that 153 (33.6%) individuals had a g-tube surgery at baseline or during the study period. The need for invasive g-tube surgery to alleviate feeding difficulties among individuals with RTT, particularly younger individuals, further underscores the considerable burden associated with this disease [21]. 

Mortality in the present study was rare and aligns with prior literature that reported death in 4.3% of individuals with classic and atypical RTT in the RNHS over a 9-year observation period, with survival exceeding 70% at 45 years of age [5]. The low mortality rates observed in this study may have been driven by the larger representation of pediatric individuals in the overall RTT sample (79.8%) and right-censoring of the RNHS data. Although death during the study was ascertained from death certificates in the RNHS [5], deaths among individuals who withdrew from the study or that occurred after the completion of the study were not systematically captured. As such, mortality rates from this study may be underestimated. Future studies could assess cause-specific mortality in RTT, which is often unknown but has been presumed to be related to cardio-respiratory issues [5], and evaluate the potential value of novel treatments for addressing events that are known drivers of mortality in RTT, such as aspiration. Together, our findings underscore an unmet need for improved symptom management earlier on in the lifespan of individuals with RTT to reduce the burden of this disease and potentially increase survival at older ages. Future studies could assess how novel treatments may impact healthcare resource use and mortality among individuals with RTT, and whether these outcomes vary by type of RTT or individuals’ age.

A novel aspect of this study was the assessment of the change in RTT severity per year. Among the overall study sample and in individuals with classic RTT, motor-behavioral dysfunction significantly increased per year. Furthermore, both clinical severity and motor-behavioral dysfunction significantly increased per year among pediatric individuals with RTT. Although not unexpected, these findings highlight the increasing burden of RTT over time. Prior literature investigating individuals with RTT aged 5–18 years in the RNHS identified a significant association between clinical impairment in RTT, as measured by the CSS and MBA, and poor physical QoL [6], suggesting that increasing clinical severity and motor-behavioral dysfunction have important negative impacts on individual’s QoL. Due to missing data for clinical measures, longer-term assessments of CSS and MBA scores among individuals with RTT were not feasible, and the annual change in RTT severity estimated by our models was limited to 2 years of data. While our findings can elucidate on the short-term changes in RTT severity, an understanding of the long-term trends in clinical severity and motor-behavioral dysfunction is limited, as developmental changes in RTT are generally slow [6]. Future studies may expand upon this analysis by investigating the annual changes in RTT severity over longer periods of time and using other clinical measures, such as the Rett Syndrome Behavior Questionnaire (RSBQ) to assess neurobehavioral severity [22]. Nonetheless, these findings provide additional insight on the changes in RTT severity in the short-term, and how these changes may vary across RTT type and individual’s age.

The findings from our study should be interpreted considering some limitations. First, as most RTT-related outcomes and therapies evaluated in this study were reported by caregivers, there is a potential for misspecification of endpoints, given the subjectivity in their assessment. However, estimates reported in this study are corroborated by published RTT literature, suggesting that our findings are representative of the real world. Second, our assessments were limited to the subset of individuals with complete information; the high proportion of missingness observed for certain variables could lead to uncertainty in the estimation of endpoints evaluated in this study. Third, the MBA scale has not been validated in individuals with RTT and as such, the reliability, validity, and relevance of this scale in the RTT population have not been established. Future research assessing motor-behavioral dysfunction in individuals with RTT using a validated instrument are warranted.

To our knowledge, this study was the first to assess changes in RTT severity over time and provide novel insights on the short-term trends of this disease in classic and atypical RTT as well as in pediatric and adult individuals. This study used data from the largest registry database specifically designed to collect data on individuals with RTT in the US. The database is uniquely rich in endpoints, such as CSS and MBA scores, allowing for a more comprehensive overview of the treatment and disease journey that might otherwise not be observed in other data sources where data capture may be less complete. Moreover, the results are likely to be generalizable to the broader RTT population. Additionally, the RHNS database has a comprehensive capture of the clinical features of RTT and over-the-counter pharmacologic therapies, which may not be captured in other data sources that necessitate a healthcare encounter or a drug prescription/dispensing, respectively, to be recorded in the database.

## Conclusions

Findings from this study highlight the considerable burden of RTT across classic and atypical RTT as well as pediatric and adult individuals. Reliance on supportive therapies and healthcare resources to manage debilitating symptoms of RTT was common in the overall study sample and more pronounced in pediatric individuals than adult individuals, highlighting the importance of early interventions to facilitate the long-term management of RTT. Despite the use of pharmacological and supportive therapies, individuals with RTT experience increasing severity of disease with respect to motor-behavioral dysfunction, underscoring the present unmet needs of this population. Future studies may provide additional insights by investigating disease management strategies in the context of novel targeted therapies and elucidate the impact of the evolving treatment landscape on the severity of RTT as well as the QoL of individuals with RTT and their caregivers.

### Electronic supplementary material

Below is the link to the electronic supplementary material.

Supplementary Material 1

## Data Availability

The data that support the findings of this study are available from the International Rett Syndrome Foundation, but restrictions apply to the availability of these data, which were used under license for the current study, and therefore, are not publicly available. Data are however available from the authors upon reasonable request and with permission of the International Rett Syndrome Foundation.

## Abbreviations

CI: Confidence interval
CSS: Clinical Severity Scale
FDA: Food and Drug Administration
g-tube: Gastrostomy tube
LRTI: Lower respiratory tract infection
MBA: Motor Behavioral Assessment
QoL: Quality of life
RNHS: Rett Syndrome Natural History Study
RSBQ: Rett Syndrome Behavior Questionnaire
RTT: Rett syndrome
SD: Standard deviation
US: United States
