# Supplementary material for: Characterizing the journey of Rett syndrome among females in the United States: a real-world evidence study using the Rett syndrome natural history study database
Source: J Neurodev Disord. 2024 Jul 26;16:42. doi: 10.1186/s11689-024-09557-6 (PMC11282812; doi:10.1186/s11689-024-09557-6)
Supplement: Supplementary file 1 — Supplementary Material 1 [file 11689_2024_9557_MOESM1_ESM.docx]

Characterizing the Journey of Rett Syndrome Among Females in the United States: A Real-World Evidence Study Using the Rett Syndrome Natural History Study Database

Damian May, PharmD, MBA; Kalé Kponee-Shovein, ScD, MS, MPH; Jeffrey L. Neul, MD, PhD; Alan K. Percy, MD; Malena Mahendran, MSc; Nathaniel Downes, BA; Grace Chen, MPH; Talissa Watson, MSc; Dominique C. Pichard, MD; Melissa Kennedy, MHA; Patrick Lefebvre, MA

Supplementary Material

Supplementary Table 1. Supportive Therapies Used among Females with RTT, Overall and Stratified by RTT Type and Age

| **Supportive therapies** | **Overall RTT**  **Cohort** | **Stratification by RTT type** | | | |  | **Stratification by age** | | | |  |
| --- | --- | --- | --- | --- | --- | --- | --- | --- | --- | --- | --- |
|  |  | **Classic RTT** | **Atypical RTT** | **Test statistic** | ***p-*value** |  | **Pediatric  (<18 years of age)** | **Adult  (≥18 years of age)** | **Test statistic** | ***p-*value** |  |
|  | **(N = 455)** | **(N = 412)** | **(N = 43)** |  |  |  | **(N = 363)** | **(N = 92)** |  |  |  |
| **Follow-up period, years, mean ± SD [median]** | 4.1 ± 1.0 [4] | 4.1 ± 1.0 [4] | 4.1 ± 1.2 [4] | -0.2 | 0.833 |  | 4.1 ± 1.1 [4] | 4.2 ± 0.8 [4] | 1.5 | 0.140 |  |
| Physical therapy, n (%) | 354 (77.8) | 323 (78.4) | 31 (72.1) | 0.9 | 0.344 |  | 317 (87.3) | 37 (40.2) | 94.3 | <0.001* |  |
| Speech-language therapy, n (%) | 337 (74.1) | 307 (74.5) | 30 (69.8) | 0.5 | 0.499 |  | 315 (86.8) | 22 (23.9) | 151.0 | <0.001* |  |
| Occupational therapy, n (%) | 321 (70.5) | 290 (70.4) | 31 (72.1) | 0.1 | 0.815 |  | 298 (82.1) | 23 (25.0) | 115.2 | <0.001* |  |
| Scoliosis treatment, n (%) | 148 (32.5) | 139 (33.7) | 9 (20.9) | 2.9 | 0.088 |  | 125 (34.4) | 23 (25.0) | 3.0 | 0.084 |  |
| Applied behavior analysis or other behavioral therapy, n (%) | 48 (10.5) | 44 (10.7) | 4 (9.3) | 0.1 | 1.000 |  | 45 (12.4) | 3 (3.3) | 6.5 | 0.011* |  |
| Vision therapy, n (%) | 20 (4.4) | 19 (4.6) | 1 (2.3) | 0.5 | 0.709 |  | 20 (5.5) | 0 (0.0) | 5.3 | 0.019* |  |
| Feeding assistance (including feeding tube), n (%) | 8 (1.8) | 8 (1.9) | 0 (0.0) | 0.8 | 1.000 |  | 8 (2.2) | 0 (0.0) | 2.1 | 0.368 |  |
| **Abbreviations:** RTT: Rett syndrome; SD: standard deviation. | | | | | | | | | | | |

Supplementary Table 2. RTT-Related Outcomes among Females with RTT, Overall and Stratified by RTT Type and Age

| **RTT-related outcomes** | **Overall RTT**  **Cohort** | **Stratification by RTT type** | | | |  | **Stratification by age** | | | | |
| --- | --- | --- | --- | --- | --- | --- | --- | --- | --- | --- | --- |
|  |  | **Classic RTT** | **Atypical RTT** | **Test statistic** | ***p-*value** |  | **Pediatric  (<18 years of age)** | **Adult  (≥18 years of age)** | **Test statistic** | ***p-*value** |  |
|  | **(N = 455)** | **(N = 412)** | **(N = 43)** |  |  |  | **(N = 363)** | **(N = 92)** |  |  |  |
| **Follow-up period, years, mean ± SD [median]** | 4.1 ± 1.0 [4] | 4.1 ± 1.0 [4] | 4.1 ± 1.2 [4] | -0.2 | 0.833 |  | 4.1 ± 1.1 [4] | 4.2 ± 0.8 [4] | 1.5 | 0.140 |  |
| Hospital or emergency room visit, n (%) | 203 (44.6) | 194 (47.1) | 9 (20.9) | 10.8 | 0.001* |  | 176 (48.5) | 27 (29.3) | 10.9 | 0.001* |  |
| Individuals without a baseline G-tube surgery, n (%) | 350 (76.9) | 314 (76.2) | 36 (83.7) | 1.2 | 0.266 |  | 275 (75.8) | 75 (81.5) | 1.4 | 0.241 |  |
| G-tube surgery | 48 (13.7) | 44 (14.0) | 4 (11.1) | 0.2 | 0.800 |  | 46 (16.7) | 2 (2.7) | 9.8 | 0.002* |  |
| Death, n (%) | 3 (0.7) | 3 (0.7) | 0 (0.0) | 0.3 | 1.000 |  | 1 (0.3) | 2 (2.2) | 4.0 | 0.105 |  |
| **Abbreviations:** RTT: Rett syndrome; SD: standard deviation. | | | | | | | | | | |  |
| **Note:** | | | | | | | | | | |  |
| 1. G-tube surgeries included endoscopic gastrostomy, gastrostomy with fundoplication, and gastrostomy without fundoplication. | | | | | | | | | | |  |

Supplementary Table 3. Annual Change in Clinical Severity Score among Females with RTT, Overall and Stratified by RTT Type and Age

| **CSS measures** | **Overall RTT**  **Cohort** | **Stratification by RTT type** | | | |  | **Stratification by age** | | | |
| --- | --- | --- | --- | --- | --- | --- | --- | --- | --- | --- |
|  |  | **Classic RTT** | **Atypical RTT** | **Test statistic** | ***p*-value** |  | **Pediatric  (<18 years of age)** | **Adult  (≥18 years of age)** | **Test statistic** | ***p*-value** |
|  | **(N = 455)** | **(N = 412)** | **(N = 43)** |  |  |  | **(N = 363)** | **(N = 92)** |  |  |
| **Follow-up period, years, mean ± SD [median]** | 4.1 ± 1.0 [4] | 4.1 ± 1.0 [4] | 4.1 ± 1.2 [4] | -0.2 | 0.833 |  | 4.1 ± 1.1 [4] | 4.2 ± 0.8 [4] | 1.5 | 0.140 |
| ***Baseline*** |  |  |  |  |  |  |  |  |  |  |
| Individuals with CSS measurement, n (%) | 454 (99.8) | 411 (99.8) | 43 (100.0) |  |  |  | 362 (99.7) | 92 (100.0) |  |  |
| CSS, mean ± SD [median] | 22.7 ± 8.0 [22] | 23.3 ± 7.6 [23] | 17.2 ± 9.7 [14] | -4.0 | <0.001* |  | 22.3 ± 7.6 [22] | 24.3 ± 9.3 [23] | 2.0 | 0.052 |
| ***Year 1*** |  |  |  |  |  |  |  |  |  |  |
| Individuals with CSS measurement, n (%) | 218 (47.9) | 198 (48.1) | 20 (46.5) |  |  |  | 207 (57.0) | 11 (12.0) |  |  |
| CSS, mean ± SD [median] | 22.0 ± 7.4 [22] | 22.5 ± 6.8 [22] | 16.8 ± 10.5 [14] | -2.4 | 0.026* |  | 21.7 ± 7.4 [21] | 26.6 ± 6.3 [29] | 2.2 | 0.031* |
| ***Year 2*** |  |  |  |  |  |  |  |  |  |  |
| Individuals with CSS measurement, n (%) | 305 (67.0) | 279 (67.7) | 26 (60.5) |  |  |  | 241 (66.4) | 64 (69.6) |  |  |
| CSS, mean ± SD [median] | 23.2 ± 8.0 [23] | 23.8 ± 7.6 [23] | 16.8 ± 10.0 [12] | -3.5 | 0.002* |  | 23.1 ± 7.8 [23] | 23.7 ± 9.0 [22] | 0.6 | 0.582 |
| **Abbreviations:** CSS: Clinical Severity Score; RTT: Rett syndrome; SD: standard deviation. | | | | | | | | | | |

Supplementary Table 4. Annual Change in Motor Behavioral Assessment among Females with RTT, Overall and Stratified by RTT Type and Age

| **MBA measures** | **Overall RTT**  **Cohort** | **Stratification by RTT type** | | | |  | **Stratification by age** | | | |  |
| --- | --- | --- | --- | --- | --- | --- | --- | --- | --- | --- | --- |
|  |  | **Classic RTT** | **Atypical RTT** | **Test statistic** | ***p*-value** |  | **Pediatric  (<18 years of age)** | **Adult  (≥18 years of age)** | **Test statistic** | ***p*-value** | |
|  | **(N = 455)** | **(N = 412)** | **(N = 43)** |  |  |  | **(N = 363)** | **(N = 92)** |  |  |  |
| **Follow-up period, years, mean ± SD [median]** | 4.1 ± 1.0 [4] | 4.1 ± 1.0 [4] | 4.1 ± 1.2 [4] | -0.2 | 0.833 |  | 4.1 ± 1.1 [4] | 4.2 ± 0.8 [4] | 1.5 | 0.140 | |
| ***Baseline*** |  |  |  |  |  |  |  |  |  |  | |
| Individuals with MBA measurement, n (%) | 453 (99.6) | 410 (99.5) | 43 (100.0) |  |  |  | 361 (99.4) | 92 (100.0) |  |  | |
| MBA, mean ± SD [median] | 46.7 ± 13.4 [46] | 47.8 ± 12.4 [47] | 36.5 ± 17.7 [36] | -4.1 | <0.001* |  | 45.8 ± 12.5 [45] | 50.5 ± 15.9 [50] | 2.7 | 0.009* | |
| ***Year 1*** |  |  |  |  |  |  |  |  |  |  | |
| Individuals with MBA measurement, n (%) | 214 (47.0) | 194 (47.1) | 20 (46.5) |  |  |  | 204 (56.2) | 10 (10.9) |  |  | |
| MBA, mean ± SD [median] | 46.0 ± 11.8 [47] | 47.2 ± 10.3 [47] | 34.9 ± 18.6 [39] | -2.9 | 0.009* |  | 45.6 ± 11.6 [46] | 53.3 ± 15.5 [56] | 2.0 | 0.046* | |
| ***Year 2*** |  |  |  |  |  |  |  |  |  |  | |
| Individuals with MBA measurement, n (%) | 298 (65.5) | 272 (66.0) | 26 (60.5) |  |  |  | 234 (64.5) | 64 (69.6) |  |  | |
| MBA, mean ± SD [median] | 48.5 ± 13.4 [48] | 49.7 ± 12.4 [49] | 35.5 ± 16.5 [37] | -4.3 | <0.001* |  | 47.8 ± 13.2 [48] | 50.7 ± 14.0 [49] | 1.5 | 0.126 | |
| **Abbreviations:** MBA: Motor Behavioral Assessment; RTT: Rett syndrome; SD: standard deviation. | | | | | | | | | | | |
